# Analyzing metaphor patterns in COVID-19 news pictures: A critical study in China

**DOI:** 10.1371/journal.pone.0297336

**Published:** 2024-02-02

**Authors:** Fu Feifei

**Affiliations:** 1 Zhejiang Gongshang University Hangzhou College of Commerce, Hangzhou, P. R. China; 2 Department of English and Communication, The Hong Kong Polytechnic University, Kowloon, Hong Kong SAR, P. R. China; University of Ghana, GHANA

## Abstract

Drawing on Critical Metaphor Analysis, this study investigates major metaphors of the COVID-19 pandemic used by the Chinese government in the specific genre of news picture. It examines patterns of metaphor use in the first year of the pandemic in China and explains how and why the government employs the identified metaphors in the Chinese political context. Results reveal that pandemic metaphors (45%) are not as widely used in news pictures as presumed, the vast majority (95%) are rendered in verbal mode, and the most salient metaphors used in news pictures are the UP/DOWN (spatial), WAR, FAMILY, and COMPETITION metaphors. This study then addresses how COVID-19 metaphors are used in the Chinese political context and claims that the Chinese government uses specific metaphors with persuasive and ideological functions. The WAR metaphor aids comprehension of abstract concepts of the pandemic treatment, the FAMILY metaphor fosters empathy among Chinese individuals to counter blame and discrimination in society, UP/DOWN (spatial) and COMPETITION metaphors stimulate action to overcome the common “enemy.” WAR and FAMILY metaphors also contribute to the construction of a greater sense of collectivism and play a crucial role in fostering a positive national identity. Implications, limitations and some directions for future research are suggested.

## Introduction

### Background of the study

The COVID-19 pandemic has brought harm and inconveniences to people worldwide since January 2020. Once it confirmed high transmission rates of the virus, the Chinese government shut down transportation and advised citizens to stay home to avoid the disease transmission. During this period, people had to rely on the media to receive pandemic-related information and news reports. On February 7, 2020, People’s Daily, a Chinese state-owned newspaper, published Chinese President Xi Jinping’s statement, claiming that, following the outbreak of the COVID-19 pandemic, the Chinese government and people had launched a “People’s War” against the pandemic. Metaphors for the pandemic—for instance, the WAR metaphor—were witnessed by Chinese society in the media from that time onwards.

A metaphor is “not just in language, but in thought and action” [1] (p.3), and “the essence of metaphor is understanding and experiencing one kind of thing in terms of another” [1] (p.5). Interpreting a metaphor consists of mapping features from the source domain onto the target domain, with specific features of the target domain are highlighted during the mapping process. The comprehension of a metaphor is not only simply cognitive but also context-based [2]. Research into disease metaphors has concluded that the WAR metaphor is the most conventionally used one [3,4]; the JOURNEY metaphor is also prominent in the discourse on cancer and other fatal diseases [3,5]. Although studies on the COVID-19 pandemic have been carried out to a large extent, existing studies do not pay much attention to the use of metaphors in news pictures, which is an essential and attractive element in news reports in the digital era. Studies in journalism assert that news picture is more perceptible and memorable modality of news report when compared to text [6]. Cognitive linguistics also concludes there is an “increasing reliance on images in our civilization” [7] (p.79).

Metaphor is also recognized as a tool for politicians to address critical issues. Employing metaphors is one strategy that politicians adopt to make abstract, strange, and unfamiliar concepts more understandable. As a particular type of framing, metaphorical framing is the use of metaphorical language to influence people’s decision-making by mapping one concept to another. Metaphorical framing in political discourse has been extensively studied [8–10], and its effect on people’s opinions is well-received. According to the meta-analysis of metaphorical political discourse [11], more than 65.9% of studies on metaphorical framing in political discourse were conducted in the United States, and the rest were conducted in European countries. Most Chinese scholars tend to focus on the translation of metaphors in political discourse [12]; only a few studies [12,13] in recent years have realized the importance of analyzing cognitive devices in political discourse to understand the ideologies behind them better. Therefore, we consider it crucial to investigate COVID-19 metaphors in news pictures in Chinese mainstream media. Knowing the patterns of COVID-19 metaphors in news pictures can help readers to understand more vividly how the Chinese government reacted to the pandemic, which political goals they wanted to achieve, and what social values they wanted to deliver.

### Critical metaphor analysis

To explore “the underlying intentions of the text producer and therefore serves to identify the nature of particular ideologies” [8](p.28), Charteris-Black [8] proposes the concept of Critical Metaphor Analysis in large corpora. Charteris-Black claims that critical analysis of metaphor could be done following the three steps: metaphor identification, metaphor interpretation, and metaphor explanation. Critical Metaphor Analysis integrates linguistic analysis with cognitive understanding and social insight, it helps researchers explore the purposes/functions of metaphors and hidden ideologies behind the chosen metaphors. Studies in this vein include Charteris-Black [8,9,14], Goatly [15], Musolff [16–18], and many others.

### Research questions

Drawing on Critical Metaphor Analysis, this study seeks to investigate how the Chinese government frames the COVID-19 pandemic through metaphorical descriptions in news pictures. The first research question (*RQ1*: *How did the Chinese government frame the COVID-19 pandemic through metaphors*?) is proposed. To address this research question, the study first constructs the corpus by collecting online data. The study then employs two coders to identify major metaphors and uncover the frequency of metaphor and three modes of metaphors (visual, verbal, and multimodal) in sample data.

In addition to examining the manifestation of COVID-19 metaphors in Chinese society, the study delves into how and why those metaphors were used in the Chinese political context. Therefore, the other two research questions are proposed to this purpose: *RQ2*: *How did the Chinese government employ the identified metaphors in the Chinese political context*? and *RQ3*: *Why did the Chinese government employ the identified metaphors in the Chinese political context*?

### Methods

#### Research time frame and data collection

The research time frame was set from January 21, 2020, which was the day that the Chinese epidemiologist Dr. Zhong Nanshan announced cases of human-to-human transmission of COVID-19 in China, to January 20, 2021, which was one year after the pandemic began in China. In this year, hundreds and thousands of news reports of the pandemic could be seen in mainstream media. The study stopped collecting data in January 2021 for another reason: although there were some upticks in the number of pandemic cases after January 2021, mainstream news agencies no longer reported disease-related news on a daily basis, resulting in fewer reports and news pictures about the pandemic.

Because the study examined the metaphorical discourse used by the Chinese government that express Chinese social values and ideologies, I decided to construct the corpus of data by collecting news pictures from a state-owned news agency, People’s Daily. People’s Daily functions under the leadership of the Central Committee of the Communist Party of China (CPC) and provides direct information on the policies and viewpoints of the CPC. People’s Daily established its Sina Weibo (Weibo) account in July 2012 and had gathered more than 153 million followers by October 2023.

This study collected news pictures from the Weibo account of People’s Daily [19]. Repeated and re-posted pictures from People’s Daily were taken into consideration because Weibo users are more likely to learn about a particular topic through retweets [20]. Data set consisted of news pictures that contain COVID-19 pandemic-related information or news pictures belonging to posts that contain pandemic-related information. A total of 4942 news pictures were collected as the corpus for this study.

### Data sampling

Determining the appropriate sample size is one of the most important factors in statistical analysis. A sample size that is too small will not produce reliable results or adequately represent the realities of the population under study. Following the practice of previous study [21] that investigates online data, this study employed the sample size calculator to determine the sample size needed to represent the target population (4942 news pictures). The sample size was determined based on the population size, the margin of error (i.e., confidence interval), and confidence level of research data.

The population in this study is 4942 news pictures. The sample size should be 1342, giving a confidence level of 99%, and a confident interval of 0.03. It is the number of actual news pictures needed to achieve the accuracy of the study. The sample size of 1342 is around 30% of the population of this study. Therefore, the study determined to analyze 30% of the corpus data (N = 1483) by stratified random sampling.

### Coding scheme

Coding was manually done by the author and a colleague who had been trained with knowledge of metaphor, and we manually identified domains of metaphors in the data. Because the study investigated metaphors in news pictures, we found the Visual Metaphor Identification Procedure (VISMIP) [22] to be applicable. VISMIP is an adaptation of Metaphor Identification Procedure Vrije Universiteit (MIPVU) [23] that aims to identify all lexical units in the discourse that can be associated with cross-domain mappings in conceptual structure. VISMIP guides us to examine indirect metaphor and direct metaphor in data and invites us to pay attention to incongruity or semantic tension [8] in metaphor identification.

**Fig 1:**
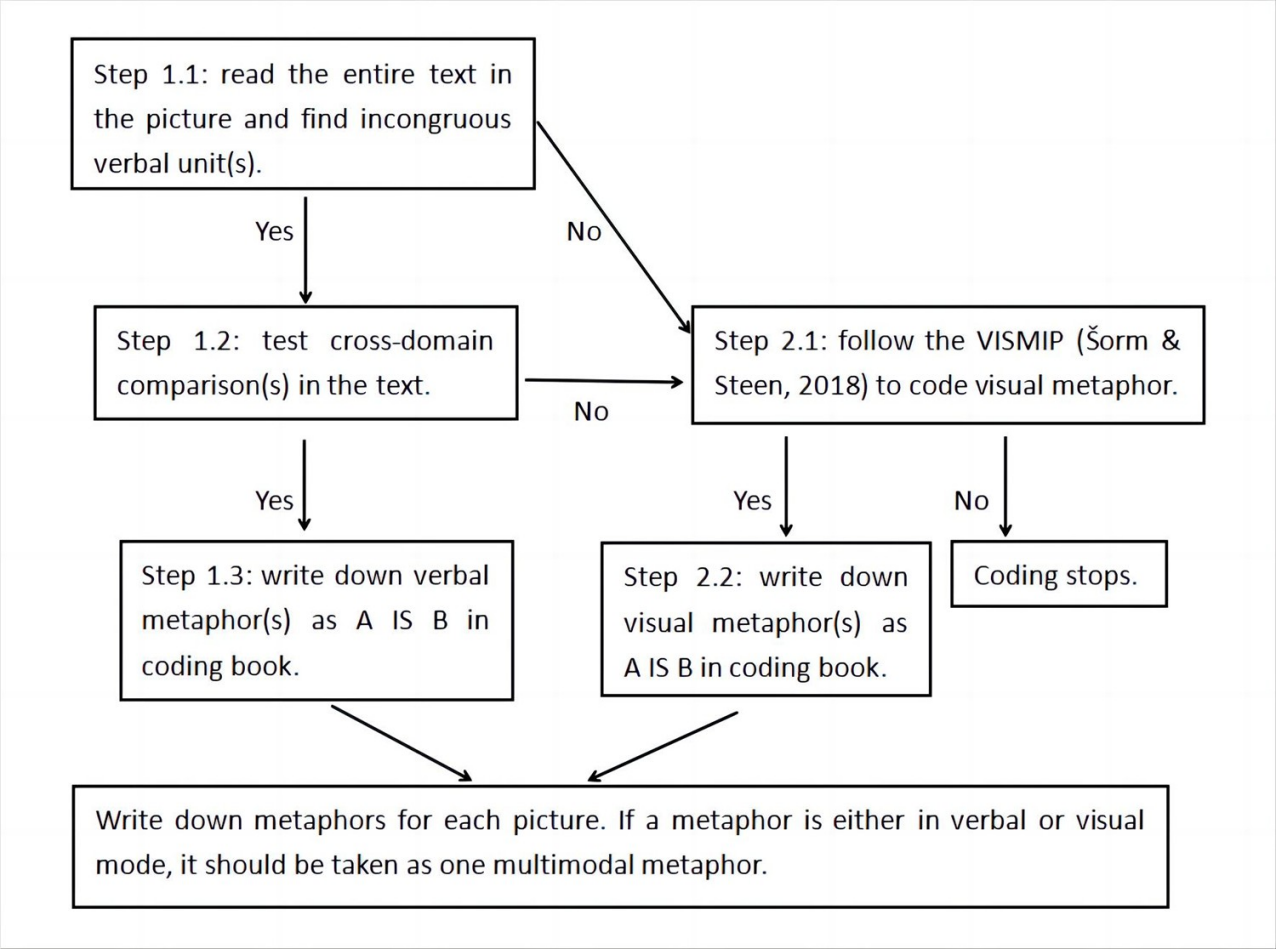
Coding procedures.

The study has slightly adjusted VISMIP in the coding scheme by first asking coders to look at incongruous verbal units. It can also facilitate a general understanding of the whole picture, which benefits our next step—visual metaphor identification. Then coders look at visual elements and follow the remaining steps of VISMIP. Finally, based on what has been identified from verbal and visual elements, coders write down what and how many metaphor(s) are in each news picture in coding books. With verbal information present in almost every news picture, our focus was not only on incongruous visual units but also on incongruous verbal units. A multimodal metaphor is one whose target and source domains are represented exclusively and predominantly in different modes [24]. During the coding process, we noted a metaphor to be a multimodal metaphor if we coded the source and target domains in different modes in the same picture. If they were exclusively verbal or visual, they were coded as verbal or visual metaphors. The coding procedures are visualized in the following Fig 1.

The coding process also includes an independent coding of 20% of the sample data (N = 1483), a check of the inter-coder reliability, coders’ independent coding of the rest of the sample data, and a discussion to reach a final agreement on coding. After both coders completed the coding and discussion, we summarized the information gathered, including the source domains, target domains, conceptual mappings, metaphor mode, and metaphor frequency in each picture.

### Inter-coder reliability

Like many other qualitative analyses, the identification of conceptual metaphors is “inevitably subjective” [25] (p.49), and the inter-coder reliability issue must be taken seriously. Šorm and Steen found it is a “challenge” [22] (p.83) to monitor the quality and reliability of VISMIP through testing whether or not VISMIP leads to sufficient agreement among analysts. Cohen’s kappa (κ) was used to measure agreements between both coders in this study. Cohen’s kappa (κ) is a robust measure of inter-coder reliability as it takes into account agreements for categorical scales that would have occurred by chance [26]. The study utilized SPSS software to compute Cohen’s kappa coefficient. All statistical analyses in this study were completed in IBM SPSS Statistics (version 26.0).

The inter-coder reliability was checked based on the independent coding of 20% of randomly selected news pictures (n = 300) before discussion. The checking of agreements (percentage and Cohen’s κ) against the total frequency of metaphorical mappings and Cohen’s κ values in both coders’ separate coding of the top 5 source domains was also completed.

In terms of agreements about the frequency of metaphors in each of the 300 news pictures, the results show that both coders reached a high percentage of agreement (95.7%). The result of the inter-coder analysis from the statistical analysis performed in SPSS is Cohen’s κ = 0.922 (*p* < 0.001), which means that both coders have reached an “almost perfect” agreement [27]. The inter-coder reliability of the coding of the top five source domains, which were UP/DOWN, WAR, FAMILY, FIGHTER, and COMPETITION, was also checked. Both coders’ coding results of frequencies of the top five source domains in the 300 news pictures were first collected. Then, the inter-coder reliability of each source domain was calculated in SPSS by statistically checking the κ value; for example, when we checked the inter-coder reliability of the coding of the source domain of UP/DOWN, we input both coders’ coding results of this specific source domain and output the κ value. The results showed that the reliability was “almost perfect” for coding the top five source domains, UP/DOWN (Cohen’s κ = 0.95), WAR (Cohen’s κ = 0.882), FAMILY (Cohen’s κ = 0.904), FIGHTER (Cohen’s κ = 0.893), and COMPETITION (Cohen’s κ = 0.909). The result of Cohen’s κ indicates that VISMIP is a reliable method of metaphor identification in this study.

## Results

To answer *RQ1*: *How did the Chinese government frame the COVID-19 pandemic through metaphors*?, this study seeks to uncover the frequency of metaphor use, variations of different modes of metaphors in news pictures, and the most saliently used source/target domains and conceptual mappings of metaphors. By doing so, it aims to help readers comprehend the patterns of COVID-19 metaphors used in the Chinese political context. To break down the analysis, the study divides RQ1 into three sub-questions and answers them in the following sections:

*RQ1a*: *To what extent did the Chinese government use metaphors in pandemic-related news pictures in a state-owned newspaper*?
*RQ1b*: *How were different metaphor modes employed in news pictures of the pandemic*?
*RQ1c*: *What were the most frequently used source domains*, *target domains*, *and domain mappings in the pictures of this news category*?

### The overall frequency of metaphor

**Fig 2:**
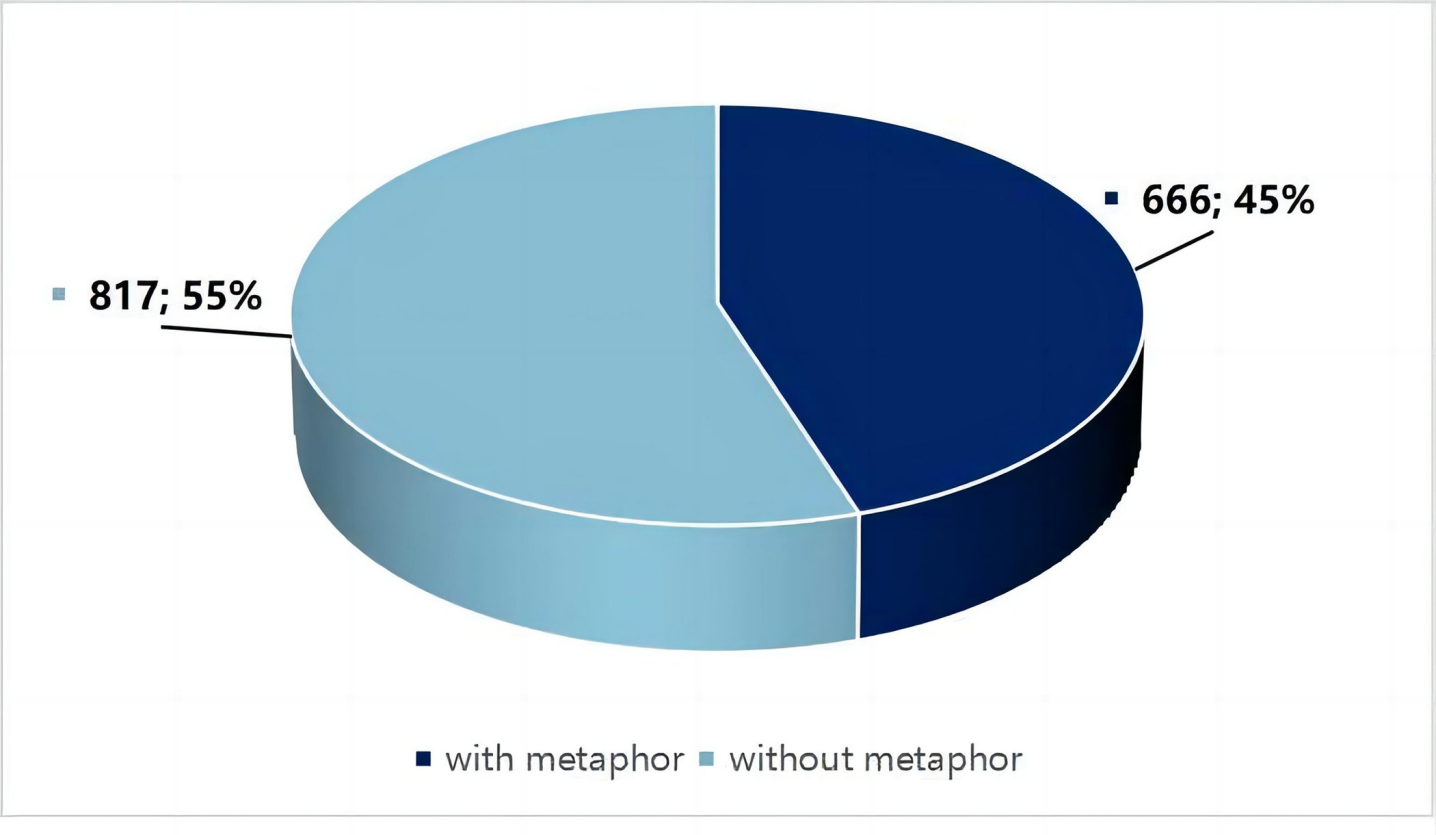
Frequency of metaphor.

To answer RQ1a (*To what extent did the Chinese government use metaphors in pandemic-related news pictures in a state-owned newspaper*?), the study presents the frequency and percentage of metaphors in the sample data. The result reveals that 666 out of 1483 news pictures (45%) contain at least one metaphor. Fig 2 illustrates the frequency and percentage of news pictures that contain metaphors and those that do not.

Although this ratio is not as high as presumed, it indicates that metaphor use is a salient language phenomenon in the Chinese political context. A binomial test also reveals that metaphors are used with statistically significant different frequencies in the sample data (*p* < .001).

### Modes of metaphor

As clarified in the last section, 666 out of 1483 news pictures contain at least one metaphor. In total, 764 metaphors were identified from the sample data. To answer RQ1b (*How were different metaphor modes employed in news pictures of the pandemic*?), we examined frequencies and percentages of verbal, visual, and multimodal metaphors among all the metaphors and also answered why certain metaphor modes were used dominantly in pandemic news pictures.

**Fig 3:**
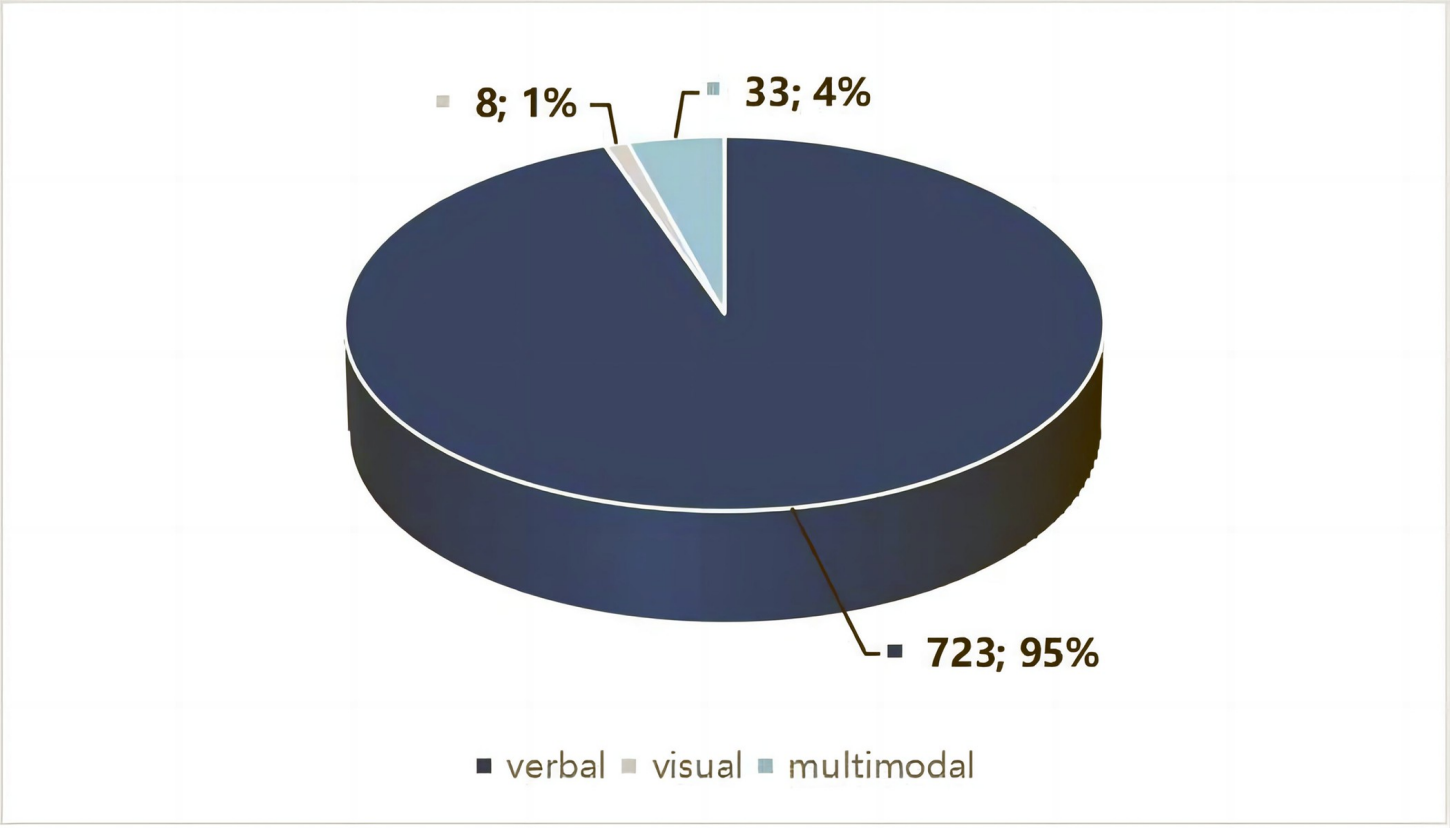
Modes of metaphor.

The result shows that among 764 metaphors, 723 (95%) are presented in verbal mode, while very few are presented in visual mode or in multimodal (8 and 33, respectively), accounting for only 5% of metaphors in the sample data. A chi-squared goodness-of-fit test reveals a statistically significant difference (*p* < .05) in the use of those three modes among 764 metaphors. Fig 3 indicates the distribution of different modes of COVID-19 metaphors in sample data.

Our results reveal that verbal metaphors are predominantly used in news pictures. The possible explanation for the dominance of verbal metaphors is that news pictures are not as well-designed as political cartoons and in the advertisement. Political cartoons are designed with specific intentions [28] that can generate ironic or humorous effects if given a second thought. In the advertisement, producers use more pictures to evoke desirable attributes associated with the product or to construct a well-connoted environment to frame the product [7], while news pictures need to be straightforward with more added verbal information.

### Major metaphors

To answer RQ1c (*What were the most frequently used source domains*, *target domains*, *and domain mappings in the pictures of this news category*?), the study presents the coding results of source and target domain frequencies for 764 metaphors in this section. This is followed by the categorization of major metaphors in the sample data.

#### Major source domains

**Fig 4:**
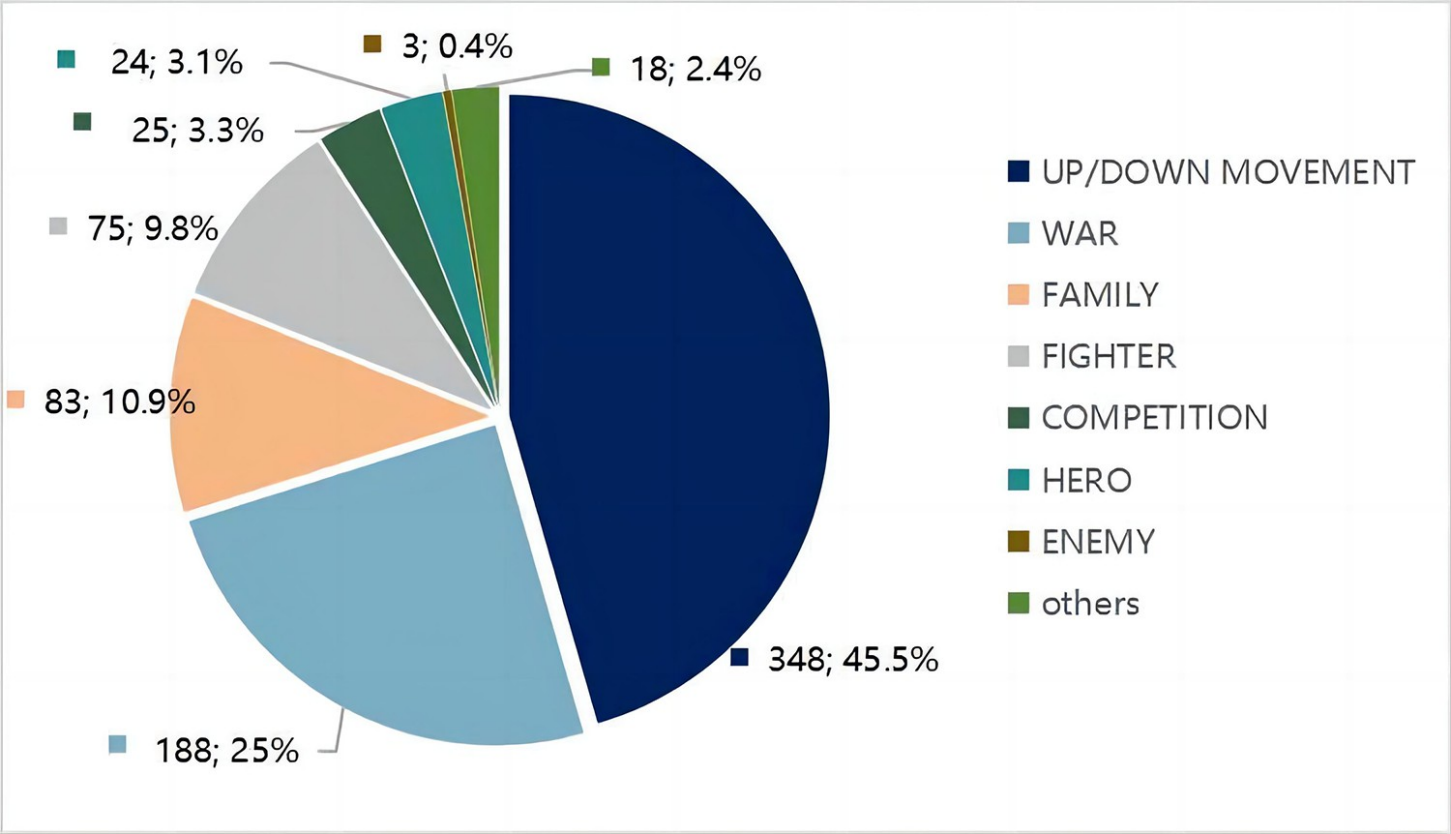
Major source domains.

The coding results shows UP/DOWN MOVERMENT, WAR, FAMILY, FIGHTER, COMPETITION, HERO, and ENEMY are major source domains in the sample data. Fig 4 reveals the coding results of source domain frequencies in sample data.

The result shows that (1) the UP/DOWN(spatial) metaphor is pervasively used since the mainstream media reported daily rises and falls in pandemic cases; (2) The WAR metaphor’s prominent use in the Chinese context’s pandemic discourse is consistent with findings from previous studies [3,4]. Scholars argue that the WAR/BATTLE metaphor is the most conventional type of metaphor used in disease discourse; (3) FIGHTER, HERO, and ENEMY metaphors are also prominent during the pandemic since they belong to the systematic WAR metaphor [29] that constitutes systemic attributes of metaphors in pandemic discourse. Cameron and colleagues [29] put forward the systematic nature of metaphor use in discourse. In this case, FIGHTER, HERO, and ENEMY metaphors combine to show that they systematically contribute to a war topic in the Chinese pandemic context; (4) The FAMILY metaphor is often seen in the research of political discourses in Western cultures [18,30]. Politicians express virtues through the FAMILY metaphor to let citizens conceptualize the nation as a family, the government as parents, and citizens as children. Its use may enhance solidarity in the public and the power of the ruling party; (5) The COMPETITION metaphor refers to comparing the treatment of COVID-19 to a competition or race. This is also highlighted in the Chinese context.

#### Major target domains

**Fig 5:**
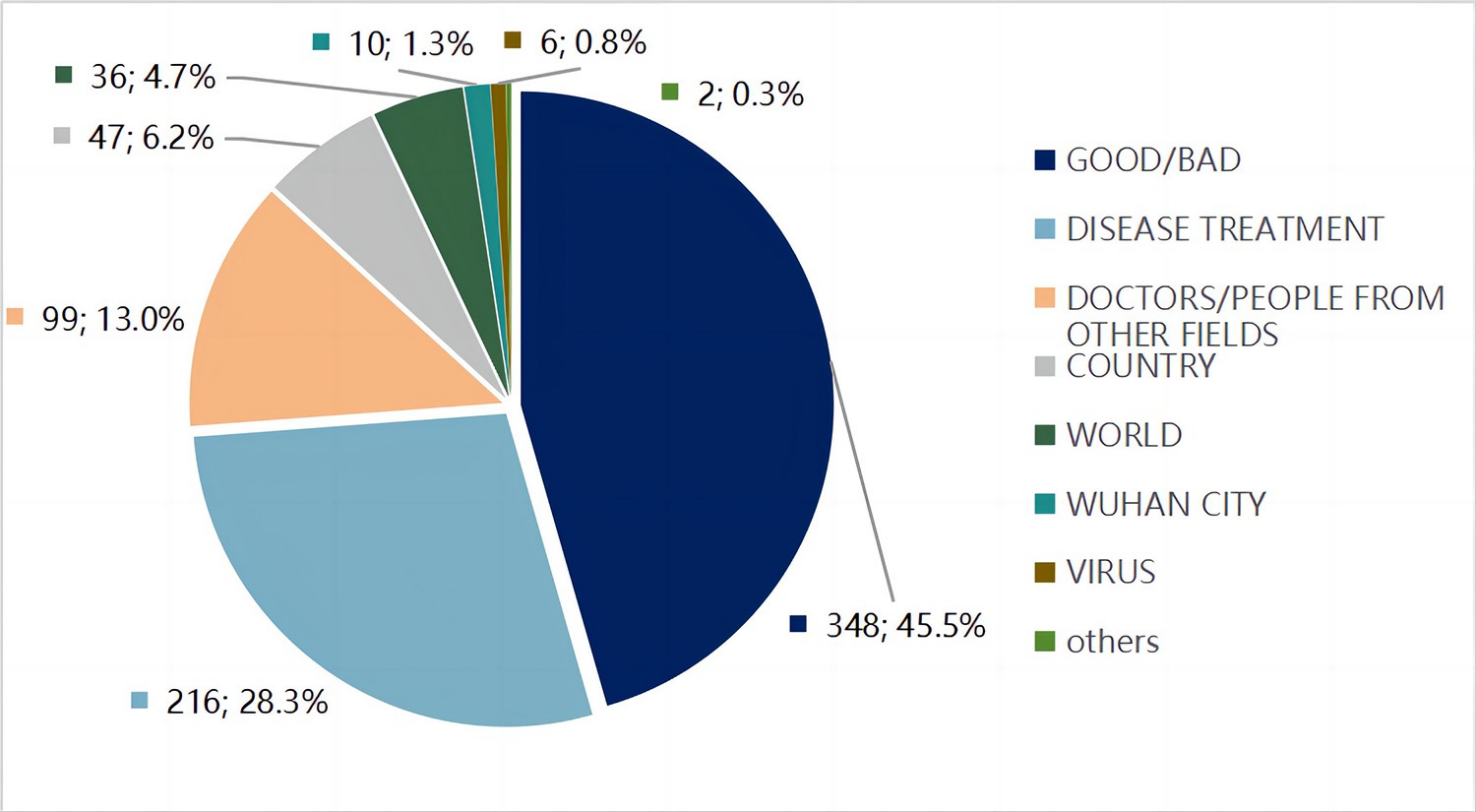
Major target domains.

The coding results indicate that the target domain of GOOD/BAD is significantly-used in reporting increases and decreases in pandemic cases. The target domain of DISEASE TREATMENT ranked the second highest on the list of target domains, because they are abstract and unfamiliar concepts to the public prior to the pandemic. Other comparisons used by the mainstream media render target domains, such as DOCTORS/PEOPLE FROM OTHER FIELDS, COUNTRY, WORLD, VIRUS, and WUHAN CITY, also prominent. Fig 5 gives major target domains identified from the sample data.

#### Major metaphors

The study then categorizes the most salient metaphors in the sample data:

1. **UP/DOWN metaphor**: GOOD IS DOWN and BAD IS UP;
2. A systematic metaphor of the **WAR metaphor**, including DISEASE TREATMENT IS A WAR, DOCTORS/PEOPLE FROM OTHER FIELDS ARE FIGHTERS, VIRUS IS AN ENEMY, and DOCTORS/PEOPLE FROM OTHER FIELDS ARE HEROES;
3. A systematic metaphor of the **FAMILY metaphor**, including THE COUNTRY IS A FAMILY, and THE WORLD IS A FAMILY;
4. **COMPETITION metaphor**: DISEASE TREATMENT IS A COMPETITION.

## Discussion

Based on the investigation on metaphor use, we have gained a clear vision that the UP/DOWN (spatial), WAR, FAMILY, and COMPETITION metaphors are four major metaphors used in the Chinese political context. This study is also concerned with the social roles that metaphors play in Chinese society and their rhetorical purposes [31] to answer *RQ2*: *How did the Chinese government employ the identified metaphors in the Chinese political context*? and *RQ3*: *Why did the Chinese government employ the identified metaphors in the Chinese political context*?

In this section, the study presents a sub-section of “Metaphor interpretation” to demonstrate how the Chinese government employed specific metaphors and a sub-section of “Metaphor explanation” to explain the functions of those identified metaphors in the Chinese political context.

### Metaphor interpretation

#### UP/DOWN metaphor

Some of the metaphors associated with spatial orientation, up-down, in-out, and front-back, are called spatial/orientational metaphors [1,32]. For example, we can conceptualize a mapping from HAPPY to UP in English expressions like “I am feeling up today” to generate the metaphor HAPPY IS UP. The spatial metaphor “does not structure one concept in terms of another but instead organizes a whole system of concepts with respect to one another” [1] (p.15) when compared to a structural metaphor that has a certain concept metaphorically structured in terms of another.

**Fig 6:**
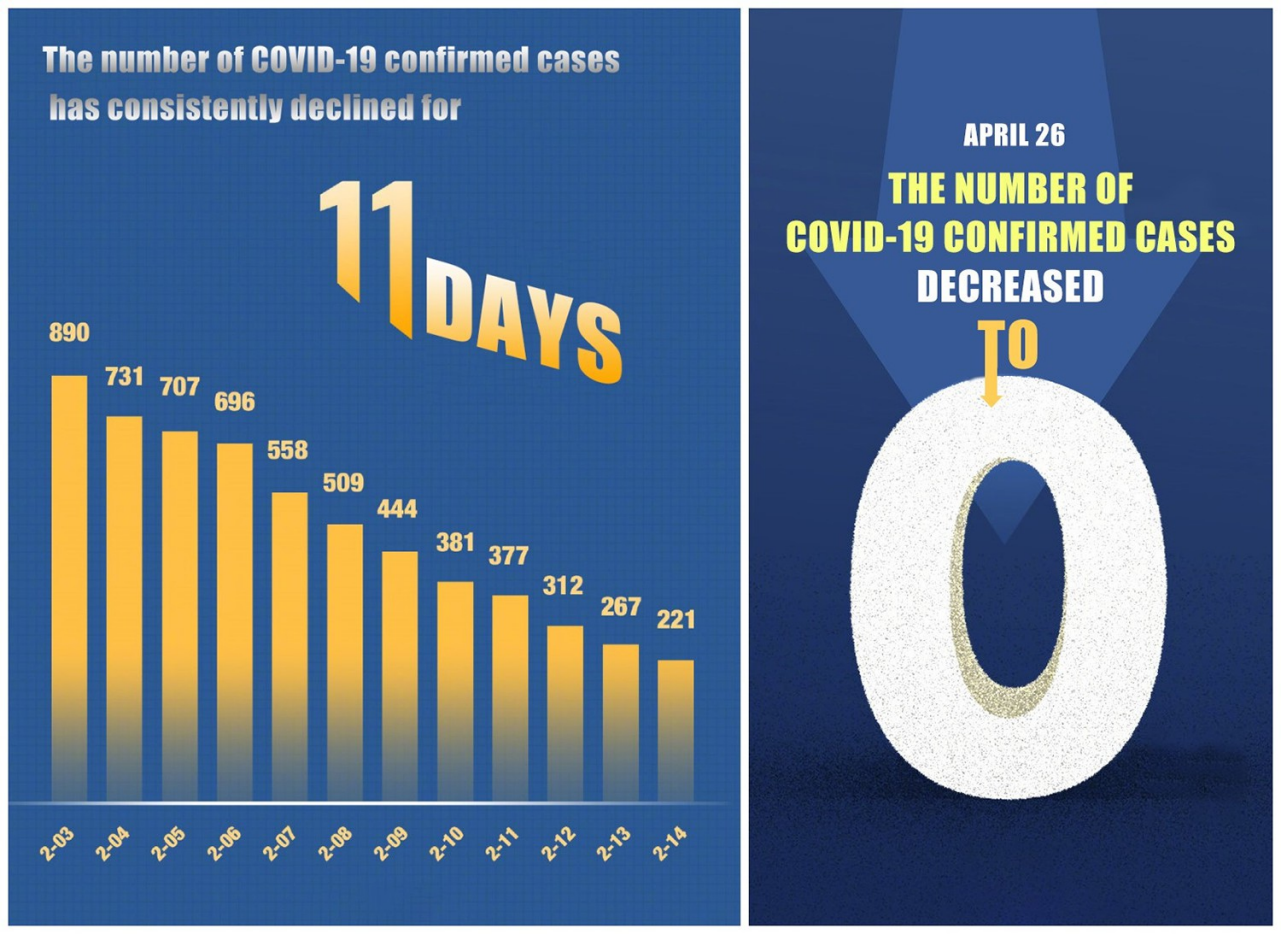
GOOD IS DOWN, and BAD IS UP. Figs 6–13 are similar but not identical to the original image, and are for illustrative purposes only.

While seeking to understand spatial metaphors, people should not merely rely on their knowledge of the physical environment; importantly, the comprehension can vary from culture to culture and from context to context. In some cases, scholars find that negative verbs may also provide a positive evaluation. For example, Charteris-Black and Musolff [33] have found in their research on Euro trading that the choice of verbs that provide a negative evaluation of a downward movement of the Euro implied a covert positive evaluation of the currency itself. The same is true in pandemics or other disease discourses. In addressing the rise and fall of confirmed cases, the use of negative verbs, DROP OF CURVE, or DECREASE IN THE NUMBER may indicate a positive evaluation of the pandemic situation, thus generating many metaphors like GOOD IS DOWN and BAD IS UP. The news pictures in Fig 6 show us how the UP/DOWN metaphor works.

In the left picture of Fig 6, the bar graph shows the decrease in the number of confirmed cases of COVID-19. This picture informs the public of the current situation of the pandemic and warn them to take careful precautions against the virus. A very big “zero” displayed in the news picture on the right side tells that there is zero case of the pandemic in Chinese society. Size is commonly used to indicate the relative salience or importance of the various elements [34] (p.85). The large size of “zero” in this picture gives people a feeling that control of the pandemic is a matter of vital importance.

These pictures help us to generate metaphors like GOOD IS DOWN and BAD IS UP. They also reveal that the government adopted the CONTROL IS GOOD metaphor throughout the pandemic. In our data, we find more than one thousand pictures that reports a rise or drop in the pandemic cases. The Chinese government reports the number of confirmed cases daily to inform and alarm people during the pandemic. It gives people a sense of urgency when the number increases, and vice versa; thus, repeated presentation of these pictures may affect ordinary people, urging them to be more self-disciplined during the pandemic. The finding of heavy use of spatial metaphors in the COVID-19 reportage draws a different conclusion from other research that mainly examines texts or verbal metaphors. Additionally, using GOOD IS DOWN and BAD IS UP metaphors in reporting COVID-19 cases generates a different view from Lakoff and Johnson’s [1] argument for the conceptual metaphors MORE IS UP, and GOOD IS UP, and LESS IS DOWN, and BAD IS DOWN.

#### WAR metaphor

The WAR metaphor as seen in Chinese news reports is a systematic metaphor for disease discourse. The study employs systematic metaphor analysis to identify patterns of metaphorical usage within the discourse, thereby contributing to a deeper comprehension of the discourse event. WAR, FIGHTER, ENEMY, and HERO metaphors combine to represent a systematic metaphor of WAR in the Chinese pandemic context. Among those metaphors, DISEASE TREATMENT IS A WAR is predominantly used; overall, four metaphors are most often presented in verbal mode.

### (1) DISEASE TREATMENT IS A WAR

The first sub-category in the systematic metaphor of WAR is DISEASE TREATMENT IS A WAR. Using the WAR/BATTLE metaphor is very common in political discourse in various cultural contexts. In the past decade, Chinese society has witnessed an increasing use of the WAR metaphor in commentary on various issues, such as the anti-poverty war and the war on corruption. At the very beginning of the pandemic, President Xi Jinping has claimed that “the battle against the pandemic is a ‘People’s War.’”

**Fig 7:**
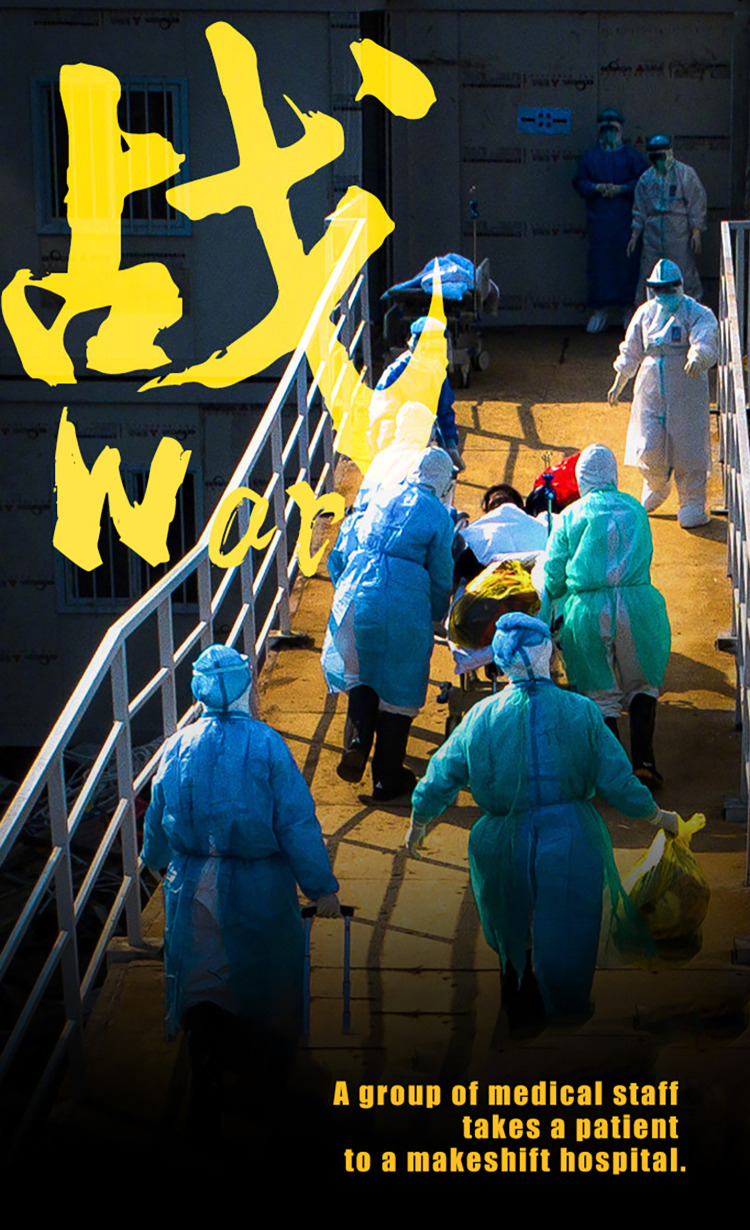
DISEASE TREATMENT IS A WAR.

Fig 7 shows an example of using the WAR metaphor in reporting on the COVID-19 pandemic in China. The picture illustrates a group of doctors who are taking a patient to the makeshift hospital. The word “war” is saliently shown in the upper left corner of this news picture. Verbal information frame the source domain of WAR. The target domain of the treatment of the pandemic can be conceptualized based on people’s clothing, and background knowledge from news reports. In the metaphor of DISEASE TREATMENT IS A WAR, the source domain of WAR is much more concrete than the target domain of the treatment of the pandemic in early 2020. The mapping brings into correspondence the elements and the relationship between the elements in the WAR domain (the source domain) with the elements and the relationship between the elements in the COVID-19 treatment domain (the target domain). This mapping in the pandemic discourse allows people to understand better how serious the situation is, thus motivating them to take urgent action against the spread of the pandemic.

The pervasiveness of WAR metaphor in the pandemic is consistent with studies in both the Western cultural context [35–37] and the Chinese cultural context [38]. The WAR metaphor is advocated and heavily used by the Chinese government. It triggers a sense of urgency in society instead of a sense of relaxation and encourages people to be motivated and take urgent action against the spread of the virus. However, we find that people hold different views toward the WAR metaphor in Western cultural contexts. For example, Sontag [39,40] criticizes the WAR/MILITARY metaphor because she thinks it has some harmful implications. Previous studies [37,41–43] have also rejected the use the WAR metaphor in relation to the COVID-19 pandemic. Additionally, scholars [3,44] have suggested using more JOURNEY metaphors than WAR metaphors in disease discourse to help patients adopt a positive attitude so they can move forward.

### (2) DOCTORS/PEOPLE FROM OTHER FIELDS ARE FIGHTERS

What the Chinese government wants people to understand is that the pandemic treatment is a “People’s War,” where both “fighters” in this “war” and people at home should face combat together and obey the rules and regulations established by the government. The “fighters” may get hurt and die from this contagious disease, and those “fighters”—which include not only doctors and other medical experts but also people from different fields, such as delivery men, community workers, and volunteers, are protecting the people and country.

**Fig 8:**
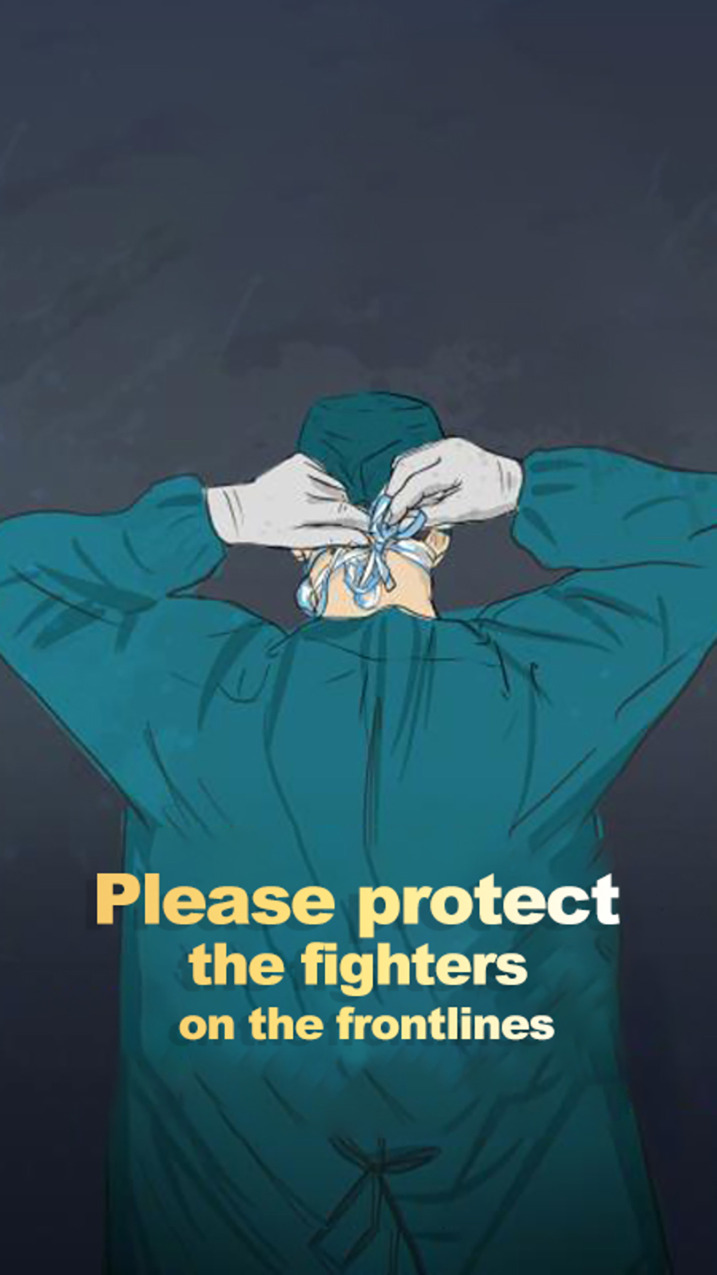
DOCTORS/PEOPLE FROM OTHER FIELDS ARE FIGHTERS.

Traditionally, fighters are those who protect the country and people. During the COVID-19 pandemic, doctors, medical experts, volunteers, community workers, and delivery workers are also fighters. Fig 8 tells us that a doctor is also a “fighter” in this “war.” The mapping in this metaphor is from medical staff to fighters. The FIGHTER metaphor allows the public to conceptualize the dangerous situation by understanding what fighters contribute during a war. To compare people to “fighters” is an alarm to other Chinese people, this helps them to understand the seriousness of the pandemic. Moreover, if the Chinese government emphasizes hundreds of times that those fighters are fighting against the pandemic, and some of them have even made sacrifices, people tend to realize those who show destructive behaviors are shameful. Then, the degree of solidarity among Chinese people may reach a higher level. The FIGHTER metaphor also gives COVID-19 patients a sense of security because it suggests that these “fighters” are all fighting for them. However, there is no comparison between patients and fighters in governmental news reports of the pandemic in China. This is different from studies in Western cultures, where people describe patients as “fighters” in a battle against their disease [3] to praise their bravery.

### (3) VIRUS IS AN ENEMY

Early confirmed cases of COVID-19 were found in the Huanan Seafood Market in Wuhan, Hubei Province. People from Wuhan, especially those who “escaped from” Wuhan in lockdown, have been delegitimated and dehumanized [45,46]. However, we find in news reports that the government asks Chinese people to be reasonable with people in Hubei. The ENEMY metaphor is employed by the Chinese government for the purpose of establishing a common “enemy” within the context of the pandemic.

**Fig 9:**
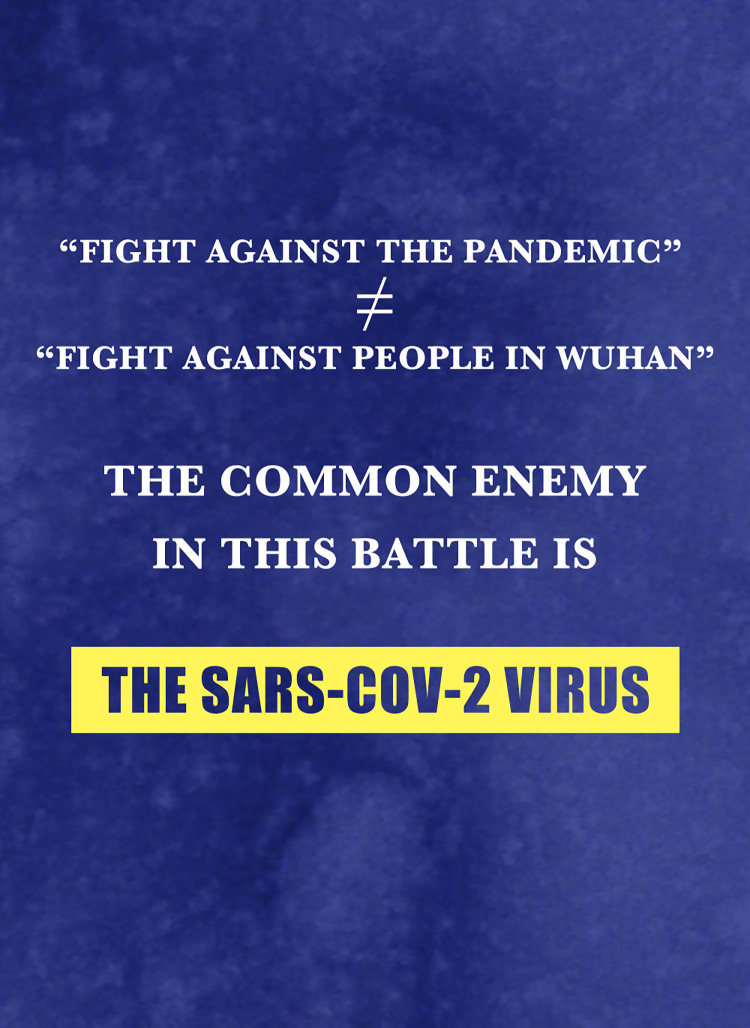
VIRUS IS AN ENEMY.

The metaphor VIRUS IS AN ENEMY is evident in Fig 9. Verbal information shows that “a fight against the disease is not a fight to people in Wuhan, Hubei Province,” instead, people should be aware that “COVID-19 is our common ‘enemy’ in this war.” Landau and colleagues [47] have addressed that using the ENEMY metaphor for skin cancer affects the degree of worry about the disease, thus making people use sunscreen as a preventative measure. In Chinese society, the ENEMY metaphor influences the degree of people’s worry about the pandemic and raises the degree of solidarity seen in combating it. People do not like to live together with an enemy. When the public come to understand that the common enemy is the virus rather than the people in Wuhan, discrimination against people in Wuhan is more or less eliminated.

### (4) DOCTORS/PEOPLE FROM OTHER FIELDS ARE HEROES

Lakoff [48] has framed “a fairy tale scenario” several times in addressing American politicians’ attitudes toward wars. In a fairy tale, there is a hero, a victim, and a villain/enemy. Lakoff claims that U.S. politicians justify their intentions to go to war by comparing the United States to a “hero” and that what the United States is doing is good for the victims. In this study, we have identified the ENEMY and HERO metaphors in the Chinese pandemic discourse.

**Fig 10:**
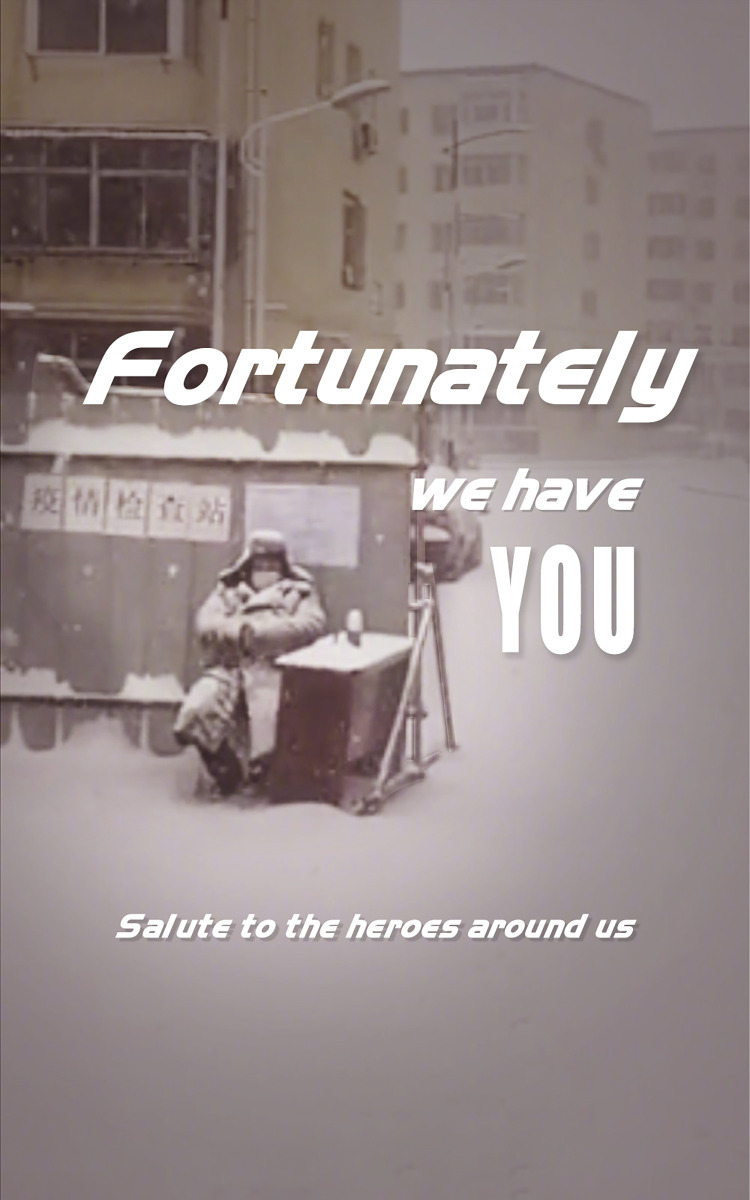
DOCTORS/PEOPLE FROM OTHER FIELDS ARE HEROES.

In Chinese society, the government considers some people as heroes in the “war,” including not only doctors but also people from other fields during the pandemic. Fig 10 shows a community worker who guards the entrance of their residential area in lockdown. Heavy snow has already covered him, but he still guards the entrance like a “hero around us.” The source domains of WAR, FIGHTER, and ENEMY conveyed heroic behavior and generated the HERO metaphor. In the sample data, we also see the Chinese government highly praises the heroic act of the city of Wuhan and the people living in Wuhan during the lockdown. Use of the HERO metaphor during the pandemic is crucial because it highlights the Chinese people’s bravery and spirit of self-sacrifice. By saying that the city and people of Wuhan are heroes and the “enemy” is the virus in this fight, discrimination against people from Wuhan, Hubei Province is eliminated, which helps to promote greater solidarity among the public.

#### FAMILY metaphor

“Family” and words from the semantic field of “family” are common in political discourse. Lakoff [30] has already found that a “strict father model” and “nurturant parents model” exist in American society, which politicians frequently adopt to address what kind of morality they want to promote. The Chinese government promotes the virtues of solidarity and friendship across the whole country by calling citizens “siblings” in a single “family.” The promotion of a “family model” in Chinese society can be witnessed in the pandemic discourse through a systematic metaphor of the FAMILY metaphor.

### (1) THE COUNTRY IS A FAMILY

Seeing that most Chinese people hold biases toward people from Hubei Province in response to the spread of COVID-19, the government uses family-related discourse in news reports to deepen the solidarity among Chinese people. The government seeks to convince people that if they already have a common “enemy”—the virus—and they need to consolidate and unite as family members in this “war.”

**Fig 11:**
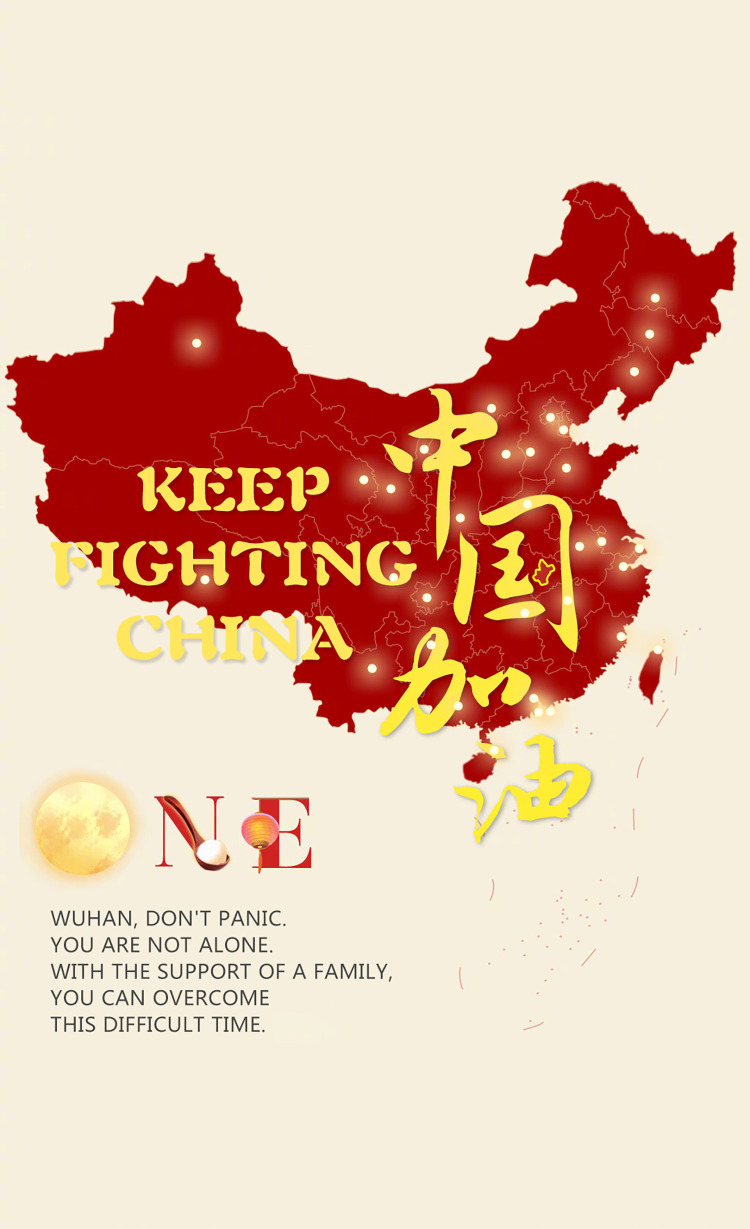
THE COUNTRY IS A FAMILY.

Fig 11 illustrates the concept of cities in China being united as “ONE” during the pandemic. Verbal information tells us that people from different cities are family. We conceptualize a mapping in this news picture from cities to family members to construct the metaphor THE COUNTRY IS A FAMILY. The FAMILY metaphor “symbolizes a source of security” [25] (p.29) and shows that China has the desire and ability to protect its family members. It expresses a sense of unity within the Chinese community to trigger people to consolidate and unite as one when facing a common enemy. Additionally, the FAMILY metaphor expresses core values in Chinese society—specifically solidarity and friendship. During the pandemic, the FAMILY metaphor has been highlighted in the Chinese context, which previous study [38] has also noticed. Nevertheless, it is not a major metaphor in Western culture.

#### (2) THE WORLD IS A FAMILY

Foreign countries suffered severely from the pandemic beginning in the late spring of 2020. Some of those countries donate medical supplies to China and China turns to offer a helping hand in response. Newspapers also report massively on the mutual help of China and those overseas countries.

**Fig 12:**
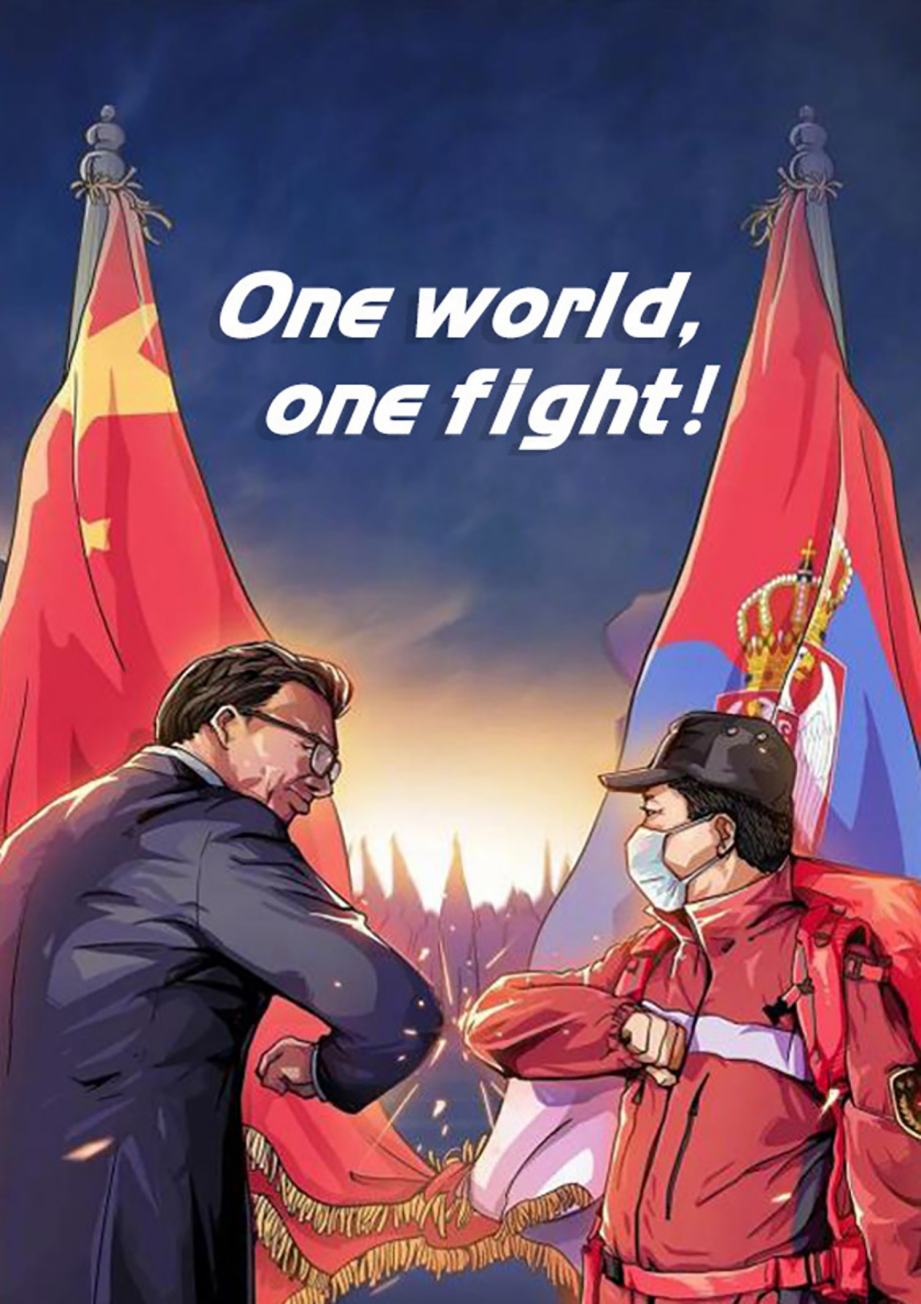
THE WORLD IS A FAMILY.

The Chinese government put forward a concept of “a global community of health for all.” China advocates that countries worldwide should work together to build a community of joint health for humankind and that people should be united as family and friends to safeguard the world’s health and security. Fig 12 shows that the Chinese government highlights the notion of “One world, one fight!”. The government emphasizes the importance of global solidarity when facing the pandemic. China has long been recognized as a responsible country, so the government acts as a family member and a close friend to those who need help to further promote this view. By using the FAMILY metaphor, China also claims its responsibilities in the pandemic.

#### COMPETITION metaphor

As believed by most philosophers and noted by Goatly, “humans are involved in a competition for survival of themselves and their progeny” [15](p.336). Human nature is competitive rather than cooperative, which result in the use of SPORT/COMPETITION/RACE metaphors in different contexts. Prior studies [49,50] have examined the COMPETITION metaphor in marketing and business contexts to find evidence that companies have highlighted the competitiveness of the business world. The source domain of COMPETITION is also commonly-used in the pandemic.

**Fig 13:**
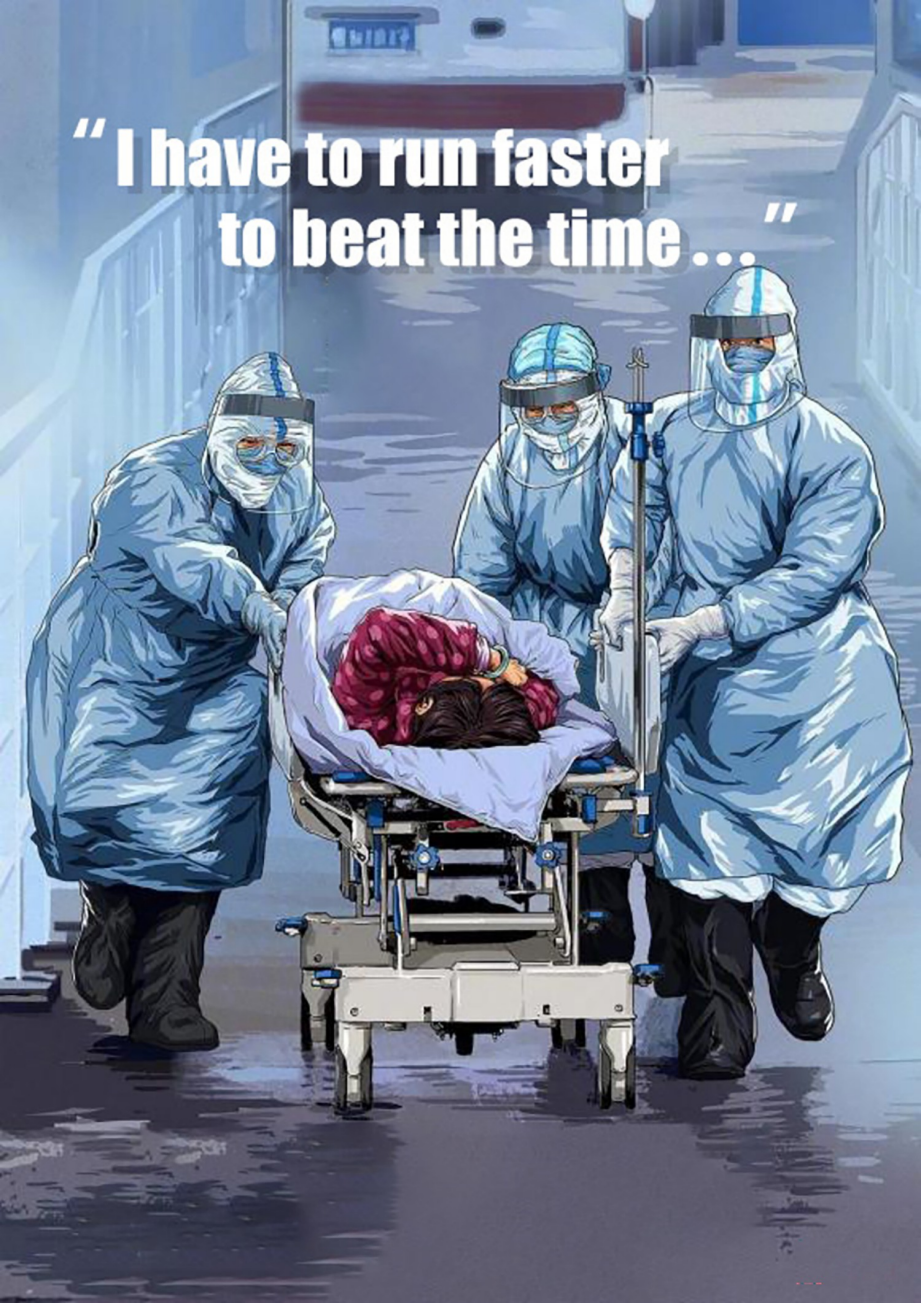
DISEASE TREATMENT IS A COMPETITION.

Fig 13 depicts the busy moment of doctors arranging patients for hospital admission. One of the doctors tells the news agency during the interview, “I have to run faster to beat the time.” The domain of a COMPETITION is mapped onto the domain of DISEASE TREATMENT in this news picture. The management of the pandemic is often described as a race/competition against time and the fast-spreading of COVID-19 in Chinese society, which promotes a competitive nature of the pandemic treatment. The COMPETITION metaphor gives doctors, patients, and the rest of the Chinese people a sense of tension and urgency. It activates a sense of urgency and urges the public to obey rules and regulations established by the government during the pandemic. The COMPETITION metaphor also forms a notion of competing against a rival, which would be the virus in the context of the pandemic.

### Metaphor explanation

In early 2020, the Chinese government and medical teams from different cities and provinces took swift action against the COVID-19 pandemic. Yet, in order to successfully manage the spread of the virus, it is imperative to communicate the severity of the situation to the public through political discourse and extensive media coverage. This study adopts the Critical Metaphor Analysis approach, which combines the cognitive linguistic view of metaphor and critical discourse analysis, to identify the hidden functions of metaphors employed by the Chinese government in the context of the pandemic discourse. Drawing on sociocultural knowledge of Chinese society, this study argues that these metaphors serve two primary functions: persuasive and ideological functions.

#### Persuasive function

Metaphors are utilized by politicians as tools for addressing issues and achieving rhetorical goals in political communication [18,25]. Mosolff [18] suggests that metaphors always have pragmatic “added value.” Metaphors can “express an evaluation of the topic, make an emotional and persuasive appeal, and/or reassure the public that a perceived threat or problem fits into familiar experience patterns and can be dealt with by familiar problem-solving strategies” [18] (p.4). Consequently, political metaphors possess “inherently persuasive power” [25].

This study contends that COVID-19 metaphors serve a persuasive function. Firstly, metaphors are employed in the pandemic context to help individuals better comprehend the abstract concepts associated with the SARS-CoV-2 virus and to realize the gravity of the pandemic during its initial outbreak. As Lakoff and Johnson [1] (p.182) assert, “metaphors may create realities for us, especially social realities.” By utilizing metaphors, the Chinese government aims to enhance understanding of the SARS-CoV-2 virus and the pandemic itself. Initially, people were unfamiliar with the SARS-CoV-2 virus. Analysis of the sample data reveals that the Chinese government utilizes metaphors comparing the pandemic to a “war” and the virus to an “enemy.” WAR and ENEMY metaphors help people understand what is happening and how they should react based on their understanding of wars.

Secondly, metaphors are employed to persuade people to reject the notion that China is to blame for the spread of the SARS-CoV-2 virus. Some politicians in the Western world blamed China excessively in early 2020. In response, the Chinese government employed THE WORLD IS A FAMILY metaphor to a large extent. The FAMILY metaphor emphasizes that the fight against the SARS-CoV-2 virus is a global endeavor, requiring international cooperation to combat the pandemic while resisting succumbing to the “political virus.” Additionally, the government cautions individuals in other provinces against blaming the people in Wuhan, Hubei Province. The ENEMY metaphor depicted in news reports shifts the focus of blame from the people in Wuhan, Hubei Province to the common “enemy”—the SARS-CoV-2 virus. Moreover, the HERO metaphor is utilized to highlight the city of Wuhan and its people as “heroes” in the fight against the pandemic, thereby combating discrimination against them. These metaphors collectively serve to dispel unfounded blame and prejudice.

Lastly, employing metaphors also serves as an alarm to the public, discouraging harmful behaviors and encouraging the adoption of protective health behaviors against the pandemic, particularly through the WAR metaphor. The use of metaphors within the WAR frame “heighten the awareness of the danger” [36] (p.51), stimulates attention and action towards the pandemic, and persuades the Chinese population to embrace protective practices. The WAR metaphor elicits a sense of urgency that captures people’s attention and motivates them to take action [4]. In this “war,” healthcare workers, medical experts, and individuals from various fields are all regarded as “fighters.” News reports employing the FIGHTER metaphor and showcasing the efforts of these “fighters” battling a common “enemy” in challenging circumstances serve to underline the seriousness of the situation. The FAMILY metaphor fosters empathy among Chinese individuals, as anyone falling ill or succumbing to the virus could be a friend or family member within a “family” scenario. UP/DOWN and COMPETITION metaphors also elicit strong responses and a collective urge to overcome the shared “enemy.”

#### Ideological function

According to van Dijk [51], ideology serves as the foundation for social representations shared by group members, enabling them to organize their beliefs and values and act accordingly. Lakoff [30] further argues that different political ideologies are constructed based on different metaphorical models in American society. Charteris-Black [31] introduces the concept of “ideological metaphor,” where metaphors express a set of beliefs and values shared by a social group and contribute to their collective worldview. Building on these ideas, this study aims to examine the ideological function of metaphors in news pictures from the state-owned news agency in the Chinese political context.

In the Chinese political context, metaphors serve a dual ideological function. Firstly, they contribute to the construction of a greater sense of collectivism. Collectivism can be understood as the prioritization of group goals and interests over individual ones. During the 19th National Congress of the CPC in 2017, President Xi Jinping emphasizes the importance of strengthening ideological and moral construction to enhance collectivist education. To align with the CPC’s mainstream ideologies, the use of the WAR metaphor in discourse promotes solidarity and collectivism among the Chinese people, especially during the COVID-19 pandemic. By portraying the fight against the virus as a common “enemy,” the media fosters empathy towards patients and people in quarantine, leading to a higher degree of societal solidarity. Similarly, the FAMILY metaphor facilitates the achievement of higher levels of solidarity by emphasizing the shared responsibility in combating the pandemic. This metaphor clarifies that the common “enemy” is the virus, not the people from Wuhan, thereby encouraging the adoption of preventive health behaviors. The depiction of healthcare workers, policemen, social workers, and volunteers as “fighters” and “heroes” on the “war” front lines further strengthens the sense of solidarity, ultimately halting the spread of the pandemic effectively in China by February 2020.

Additionally, metaphors play a crucial role in reflecting a country’s political status and in fostering a positive national identity. In Lakoff’s [30] research on political discourse, he has addressed that metaphorical reasoning is prevalent among both conservatives and liberals when discussing the relationship between the nation and its citizens. Similarly, Musolff [52] highlights the ethical dimension of metaphors in politics, evident in British and German newspaper articles on the European Union. Building on this understanding, the FAMILY metaphor in the Chinese context conveys the notion that China is a responsible country not only to its own citizens but also to the international community. By providing a sense of security and building public confidence, China projects itself as a country that actively calls for international cooperation. Through efforts focused on containing the virus within Hubei Province and limiting external spread, China assumes a decisive leadership role in affording the world time to prepare and respond to the virus. Chinese medical teams conduct research, share findings, and collaborate with the international community. Consequently, the identified metaphors in this study contribute to the construction of a Chinese national identity centered around responsibility and global cooperation.

## Conclusion

Metaphors have been pervasive in China’s media coverage of the COVID-19 pandemic since its outbreak in early 2020, but the use of metaphors in Chinese news pictures has not received enough scholarly attention. To examine the metaphorical framing of the COVID-19 pandemic in Chinese political discourse by assessing the inclusion of metaphors in news pictures, the study constructs the corpus, analyzes the most salient metaphors, and explains how and why they are used during the pandemic.

Some implications are possible in this investigation of COVID-19 metaphors in the Chinese political context. Practically, this study complements the study of COVID-19 metaphors in China. From China’s practice in combating the COVID-19 pandemic, we see the significance of examining metaphors used to conceptualize the pandemic. This study emphasizes the use of spatial metaphors in news pictures, which have not received enough attention to data from other studies. The study concludes that the Chinese government focuses on using WAR and COMPETITION metaphors to trigger a sense of urgency and on using the FAMILY metaphor to consolidate people and cultivate collectivism among the public. Nevertheless, we also note that, in Western cultural contexts, most scholars [35–37,41–43] have rejected the WAR metaphor in the pandemic while instead using the FIRE metaphor [36] or mentioning a zombie apocalypse [37]. This allows readers to rethink the appropriateness of metaphors in different cultural contexts and, once again, proves that the comprehension of metaphor is context-based [2].

Second, this investigation contributes to the existing literature by providing insights into the reasons and motivations for different metaphors in the Chinese political context. We have seen some studies examining Chinese political discourse from linguistic perspectives [12,13], but the number still needs to be increased. Metaphors not only aid in conceptualizing abstract and unfamiliar concepts but also elicit emotional responses, attention, and action among the public. In this case, metaphors act as an alarm in Chinese society, discouraging harmful behaviors and encouraging proactive measures against the pandemic. Moreover, metaphors foster empathy and solidarity among Chinese people, contributing to the national and international identity of China as a responsible country.

Theoretically, the study challenges the conventional understanding of spatial metaphors and reveals the context-dependent nature of metaphor comprehension. Contrary to the belief that GOOD IS UP and BAD IS DOWN, the study finds that spatial metaphors such as GOOD IS DOWN and BAD IS UP are generated in the discourse around COVID-19 cases. This aligns with previous research [33] on Euro trading and suggests the covert positive evaluation of downward movement. Furthermore, the study addresses the divergence in the use of metaphors in disease discourses between Western and Chinese cultures. Experimental research that examined the metaphorical perceptions of COVID-19 among social media users in Western societies has revealed that all metaphors attribute “negative meanings” [53] (p. 8). Western scholars [35–37,41–43] reject the WAR metaphor, while Chinese scholars [38,54,55] recognize it as a powerful linguistic tool. This study also demonstrates that the WAR metaphor, along with COMPETITION and FAMILY metaphors, effectively communicates the severity of the SARS-CoV-2 virus and motivates preventive actions among the Chinese population. Once again, the study emphasizes the importance of choosing appropriate metaphors for effective communication in different cultural contexts.

## Limitation and further research

As with any research endeavor, the study is not without limitations. This section aims to present limitations and suggestions for follow-up studies that may be made. First, we have examined a limited number of sample data from a single type of mainstream media in this study. Future studies might investigate more data from official and nonofficial, Chinese and overseas outlets for better generalization. Second, critical analyses of COVID-19 metaphors have been conducted, however, further research is needed to examine the impact of these metaphors on people and society. Future research could design experimental studies to investigate how metaphors influence people’s risk perception and protective health behaviors in societies.
